# Spotted Fever

**Published:** 1873-05-15

**Authors:** G. D. Mitchell

**Affiliations:** Darwin, Ill.


### SPOTTED FEVER.

BY G. D. MITCHELL, M.D., DARWIN, ILL.

READ BEFORE THE CLARK COUNTY MEDICAL SOCIETY, APRIL 2D, 1873.

None of the names, Cerebro-Spinal Meningitis, Spotted Fever, Cerebro-Spinal Fever, selected by writers to designate the nature of this disease, fully comprehend its true pathology, it appears to me. I will therefore select a name to suit my own views of its nature: Rheumatic-Malarial Cerebro-Spinal Fever appears to me to more fully describe its nature, so far as it has come under my observation and treatment. Of the particular symptoms of this disease I will not speak, as it cannot be mistaken for any other disease.

The great mistake that has been made, and is likely to be made, I think, is in the true pathology of the disease. In my opinion it is not inflammatory in its essential nature. I believe it may become inflammatory in its course, if not arrested early. It is eccentric in nature—not centric. It is systemic, and not an inflammation of the membranes of the brain and spine. It is, in my opinion, a disease of the blood or fluids of the system, septic and asthenic in nature. This fluid poison or malaria produces its effects upon the cerebro-spinal system, in combination with that state or diathesis of the system in which sub-acute rheumatism or rheumatic fever takes place. In the disease as it appeared in York and Darwin townships last winter, the exciting cause, to all appearance, was the same we generally ascribe to rheumatism. The predisposing cause would be this poisoned or septic condition of the fluids, rendered so, perhaps, by a malarial diathesis. If it is rheumatic, why not inflammatory? Is rheumatism inflammatory? Suppuration, I believe, does not result from rheumatism, as it does from all other inflammations. That it is in some way connected with a rheumatic nature is argued from the fact that nearly all the children, and many adults, in this locality, were affected with rheumatism of the muscular fibre. Pain and swelling in the arms, feet, legs, neck, back, and other parts of the body, prevailed throughout the country during the time of the greatest fatality from spotted fever. It is worthy of remark that during last winter we had very few cases of inflammatory diseases. Not a tenth part of the usual cases of pneumonia occurred in this locality last winter. That this disease is connected with a malarial influence is argued from the fact that in York township it prevailed the most extensively in the vicinity of Mill and Snider creeks, where they run into the low Wabash bottom, where malarial fevers prevailed most extensively last summer and fall. In Robinson and Oblong, in Crawford county, where this disease prevailed most fatally a few weeks before it made its appearance here, I do not know whether this will hold good. They have level clay lands; ours is a sandy, dry soil. That this disease is a blood poison is argued from the fact that most cases have the petichia, or red spots, from the very first appearance of alarming symptoms.

Spotted fever is an entirely different disease from simple idiopathic spinal meningitis, cerebro-spinal meningitis, or mytotis; they are sporadic, not epidemic. In the contraction of the muscles of the neck and back, and the mental derangement which always obtains in these sporadic cases, the symptoms are the same as we have in most cases of the so-called spotted fever.

If the disease is inflammatory, why does it not yield to antiphlogistic treatment? All cases treated as such prove fatal. Twenty-five years ago a disease very similar to this prevailed in this locality, apparently following a fatal epidemic erysipelas. Physicians at that time were firmly of the opinion that the brain symptoms were from erysipelatous inflammation of the membranes of the brain and spine. But we have had comparatively no erysipelas the past winter.

*In the treatment* of this disease all depletory and antiphlogistic remedies, as such, should be avoided. The leading indications are, first, to sustain the vital powers; second, to control the muscular contractions, hyperæsthesia, and restlessness; third, to correct the secretions.

In all cases where there are reasons to believe that the stomach and bowels are loaded with food, or an excess of bilious matter, I give a mild emeto-cathartic of calomel and ipecac, aa. ten to fifteen grains, and repeat in twenty or thirty minutes if emesis is not produced. If this does not produce purgation in a short time, give an injection of soapsuds. Whilst this is being given apply extensively, and repeatedly, mustard to the whole length of the back, to the extremities, chest, and abdomen. These should be continued till the muscular contraction and delirium are relieved. Blisters act too slowly, and appear to increase the irritability. They may be used to better purpose after all the active symptoms pass off.

Immediately after the action of the emetic, I give, to a patient twelve years old, the following:

℞.—Sulph. quinia, grs. iii. to iv.;
Sulph. Doveri, grs. vi. to viii.;
Sulph. morphia, gr. $\frac{1}{8}$;
Calomel, grs. iii.; every two, three, or four hours, according to the effect upon the pulse, and restless condition of the patient. If there are no muscular contractions, and a tendency to coma or stupor, I omit the morphia. Between each dose I give the following:

℞.—Elixer valerian ammonia, ʒ i.
Bromide potassii, grs. iii. to iv.
Tinct. gelsemium, gts. xx. to xxx.
Carbolic acid, gts. iii.

The doses of these remedies must be regulated according to their effects upon the patient. The pulse must be brought up full and strong, the muscular contraction and excessive irritability must be controlled. This can only be done by the combined effects of tonics, opiates and sedatives. Quinia alone will not answer; neither will sedatives and opiates. I give carbolic acid for its antiseptic effects. To withhold quinia and stimulants because the pulse may be from 80 to 110, and to withhold nervous and arterial sedatives because the pulse is below that point, is dangerous. Whisky may be used in the early stage, as a nerve excitant, to aid in effecting reaction; further than this its use is dangerous, because it increases the irritability and delirium. It may possibly act as an antiseptic in the course of the disease. After producing one evacuation of the bowels, keep them controlled for forty-eight hours or longer—(an excessive run upon the bowels the first two days will tend to fatal results)—continuing the calomel in small doses, with a view to its constitutional effects. This will be a safeguard to effusion upon the membranes of the brain, if there should be such a tendency. The application of boiled ears of corn to the patient's body, if not used too extensively, will assist in relieving muscular contraction, and supplying the body with heat. It is too much of a depletory remedy to be used extensively. The application of dry heat is much better. After reaction has fully taken place, and the violent cerebro-spinal symptoms are relieved, I give the following:

℞.—Tinct. colchicum, } aa. ss.,

Fl. ext. ergot, }

Iodide potassii, grs. iv., as a remedy for the rheumatic complication, still continuing the other remedies as long as there are any cerebro-spinal symptoms.

After the violent symptoms have passed off the patients are left in a prostrate and debilitated condition, for which I have used, with decided benefit, elixer calisaya bark, with strychnia, hypophosphite, iron, lime, soda, and potassa, aa. one drachm, three or four times a day. They produce a decided vitalizing effect.

In nearly all the cases convalescence is slow, attended with nervous and rheumatic pains, in the temples, neck, head, back, and limbs. The patients usually have a good appetite, but there is defective assimilation of food; remaining emaciated some times for weeks and months.

About one-third or one-fourth of the true cases proved fatal. There were many cases reported as spotted fever which were either not the disease at all, or very mild cases. It is worthy of remark that all other diseases partook somewhat of the same nature. We had an unusually large amount of malarial fevers last summer and fall ; many cases of ague continuing through the winter.

The view that this disease is rheumatic and malarial in its nature, is not entirely new with me ; it has been referred to by other writers. I give my own views, as impressed upon me after dealing with it myself. I cannot tell why it has not prevailed extensively, as it has in certain localities in this portion of the country.

